# Prevalence and distribution of glucose-6-phosphate dehydrogenase (G6PD) variants in Thai and Burmese populations in malaria endemic areas of Thailand

**DOI:** 10.1186/1475-2875-10-368

**Published:** 2011-12-15

**Authors:** Papichaya Phompradit, Jiraporn Kuesap, Wanna Chaijaroenkul, Ronnatrai Rueangweerayut, Yaowaluck Hongkaew, Rujira Yamnuan, Kesara Na-Bangchang

**Affiliations:** 1 Thailand center of Excellence on Drug Discovery and Development (TCEDD), Thammasat University (Rangsit campus), Patumthani 12121, Thailand; 2 Mae-Sot General Hospital, Mae-Sot, Tak Province, Thailand

## Abstract

**Background:**

G6PD deficiency is common in malaria endemic regions and is estimated to affect more than 400 million people worldwide. Treatment of malaria patients with the anti-malarial drug primaquine or other 8-aminoquinolines may be associated with potential haemolytic anaemia. The aim of the present study was to investigate the prevalence of G6PD variants in Thai population who resided in malaria endemic areas (western, northern, north-eastern, southern, eastern and central regions) of Thailand, as well as the Burmese population who resided in areas along the Thai-Myanmar border.

**Methods:**

The ten common G6PD variants were investigated in dried blood spot samples collected from 317 Thai (84 males, 233 females) and 183 Burmese (11 males, 172 females) populations residing in malaria endemic areas of Thailand using PCR-RFLP method.

**Results:**

Four and seven G6PD variants were observed in samples collected from Burmese and Thai population, with prevalence of 6.6% (21/317) and 14.2% (26/183), respectively. Almost all (96.2%) of G6PD mutation samples collected from Burmese population carried G6PD Mahidol variant; only one sample (3.8%) carried G6PD Kaiping variant. For the Thai population, G6PD Mahidol (8/21: 38.1%) was the most common variant detected, followed by G6PD Viangchan (4/21: 19.0%), G6PD Chinese 4 (3/21: 14.3%), G6PD Canton (2/21: 9.5%), G6PD Union (2/21: 9.5%), G6PD Kaiping (1/21: 4.8%), and G6PD Gaohe (1/21: 4.8%). No G6PD Chinese 3, Chinese 5 and Coimbra variants were found. With this limited sample size, there appeared to be variation in G6PD mutation variants in samples obtained from Thai population in different regions particularly in the western region.

**Conclusions:**

Results indicate difference in the prevalence and distribution of G6PD gene variants among the Thai and Burmese populations in different malaria endemic areas. Dosage regimen of primaquine for treatment of both *Plasmodium falciparum *and *Plasmodium vivax *malaria may need to be optimized, based on endemic areas with supporting data on G6PD variants. Larger sample size from different malaria endemic is required to obtain accurate genetic mapping of G6PD variants in Burmese and Thai population residing in malaria endemic areas of Thailand.

## Background

Glucose-6-phosphate dehydrogenase (G6PD) is a metabolic enzyme that catalyses the first reaction in the pentose phosphate pathway, providing reducing power to all cells in the form of NADPH (reduced form of nicotinamide adenine dinucleotide phosphate). NADPH enables cells to counterbalance oxidative stress triggered by oxidant agents. The G6PD gene is located at the telomeric region of the X chromosome (band Xq28), consisting of 13 exons and 12 introns. It encodes 515 amino acids and a GC-rich (more than 70%) promoter region [1].

G6PD deficiency is common in malaria endemic regions and is estimated to affect more than 400 million people worldwide. It is a hereditary genetic defect, which is one of the most prevalent polymorphisms and enzymopathies in humans, particularly in males [2-4]. This genetic defect was discovered in 1956 when some patients developed haemolytic anaemia after the dose of the anti-malarial drug primaquine [5]. G6PD-deficient erythrocytes are more susceptible to destruction by oxidative stress than normal erythrocytes due to the lower NADPH levels. Individuals with this genetic defect may exhibit non-immune haemolytic anaemia in response to a number of stimuli, most commonly infections or exposure to certain medications or chemicals [6].

The geographical distribution of malaria is remarkably similar to the world distribution of deficient G6PD variants [7]. It is postulated that the high frequency of G6PD deficiency has arisen because G6PD deficient variants confer some protection or resistance against malaria caused by *Plasmodium falciparum *and *Plasmodium vivax *[8]. Significant selective advantage against severe malaria has been reported in deficient individuals and heterozygous female carriers of deficient alleles. Malaria has also been implicated in the spreading of deficient variants in malaria endemic areas. A number of different G6PD deficient variants have reached polymorphic frequencies and each has a characteristic distribution in parts of the world where malaria is currently or was previously endemic. The mutations result in protein variants with different levels of enzyme activity that are associated with a wide range of biochemical and clinical phenotypes [9]. Approximately 140 mutations affecting the gene coding sequence have been reported [2,10], most of which are single-base substitutions leading to amino acid replacements. In Southeast Asia, a large number of G6PD deficient variants have been reported from various populations. G6PD Viangchan and G6PD Mahidol are most prevalent in Thailand [11]. G6PD-Mahidol was reported as the dominant mutation in Burmese population [12,13]. G6PD Viangchan was found to be the most common variant in Laotians [12], Malaysian Malays [14,15], Cambodians [16] and Vietnamese [17]. Apart from mutations that lead to enzyme deficiency, several polymorphic sites in introns have been identified, enabling the definition of G6PD haplotypes [2]. Since malaria and G6PD deficiency share similar geographical distribution, treatment of malaria patients with the anti-malarial drug primaquine or other 8-aminoquinolines may be associated with potential haemolytic anaemia. The aim of the present study was, therefore, to investigate the prevalence of G6PD variants in Thai population who resided in malaria endemic areas (western, northern, north-eastern, southern, eastern and central regions) of Thailand, as well as the Burmese population who resided along Thai-Burma border in Mae Sot district, Tak province, Thailand.

## Methods

### Samples

**Figure 1:**
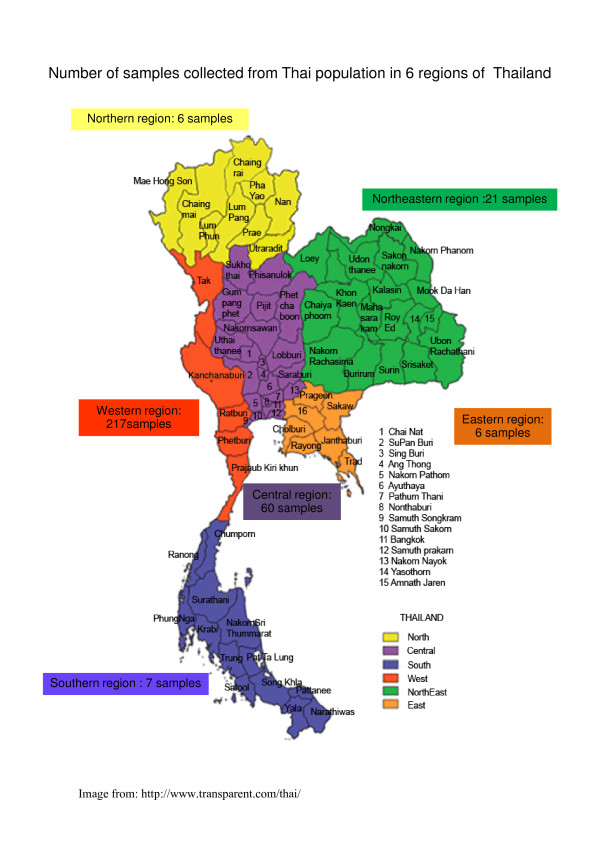
**Number of sample collected from Thai populationin 6 regions of Thailand**.

The study was conducted during the year 2008 and 2009. Blood samples (100 μl each, collected onto Whatman No. 3 filter paper) were obtained from Thai and Burmese individuals (both healthy subjects and patients with malaria) residing in malaria endemic areas of Thailand. These included a total of 317 Thai (84 males, 233 females) including north (six samples), north-east (21 samples), south (seven samples), west (217 samples), east (six samples) and central (60 samples) regions of Thailand, and 183 Burmese populations (11 males, 172 females) who had residential areas along the Thai-Myanmar border in Mae Sot district, Tak province, Thailand (Figure 1). All samples were randomly collected for genotyping of G6PD variants without any screening of G6PD deficiency. The average age of all subjects was 29 (range 15-45) years. The study was approved by Ethics Committee of Ministry of Public Health of Thailand. Informed consents for study participation were obtained from all subjects before study.

### Analysis of G6PD variants

Genomic DNA was extracted from dried blood spot samples using standard phenol - chloroform method [18] with modification. Ten common G6PD variants previously reported in Southeast Asia [19] were analysed using PCR-RFLP analysis as previously described [11,20]. These included G6PD Gaohe A95G, G6PD Chinese 4 G392T, G6PD Mahidol G487A, G6PD Chinese 3 A493G, G6PD Coimbra C592T, G6PD Viangchan G781A, G6PD Chinese 5 C1024T, G6PD Union C1360T G6PD Canton G1376T, and G6PD Kaiping G1833A.

Polymerase chain reaction was performed to generate the PCR fragments of the ten variants under 1 cycle of 5 min at 94°C, then 35 cycles of 1 min at 94°C, 1 min at 58°C, 1 min at 72°C, and final extension at 72°C for 10 min of amplification condition. PCR fragments were digested with appropriate enzymes and analysed on a 3% agarose gel containing ethidium bromide according to the method of Huang et al. and Nuchprayoon et al. [20,21]. The PCR amplification was directly performed without the screening of G6PD deficiency (Table 1).

**Table 1:** Restriction enzymes and result of genetic polymorphisms of G6PD mutation variants

G6PD Variant	cDNA Nucleotide Substitution	Amino acid Substitution	Restriction enzyme	Results (bp)
**Gaohe**	95 A > G	32 His > Arg	*Mlu*I	N 198, M 174+24

**Chinese 4**	392 G > T	131 Gly > Val	*BstE*II	N 188+15, M 203

**Mahidol**	487 G > A	163 Gly > Ser	*Hind*III	N 104, M 82+22

**Chinese 3**	493 A > G	165 Asn > Asp	*Ava*II	N 120+11, M 87+33+11

**Coimbra**	592 C > T	198 Arg > Cys	*Pst*I	N 157+83, M 157+63+20

**Viangchan**	871 G > A	291 Val > Met	*Xb*aI	N 126, M 106+20

**Chinese5**	1024 C > T	342 Leu > Phe	*Mbo*II	N 187, M 150+37

**Union**	1360 C > T	454 Arg > Cys	*Hha*I	N 142+45+27, M 187+27

**Canton**	1376 C > T	459 Arg > Leu	*Afl*II	N 214, M 194+20

**Kaiping**	1388 G > A	463 Arg > His	*Nde*I	N 227, M 206+21

*N *normal digestion result

*M *mutant digestion result

## Results

Based on PCR-RFLP analysis of ten non-synonymous G6PD mutations, 47 out of 500 samples were found to carry G6PD mutation variants. The prevalence of G6PD mutation variants in Thai and Burmese ethnics were 6.6% (21/317) and 14.2% (26/183), respectively (Table 2).

**Table 2:** The prevalence of 10 glucose -6-phosphate dehydrogenase (G6PD) variants in Thai and Burmese populations

Name of variant	cDNA Nucleotide Substitution	Amino acid Substitution	Ref SNP ID	Class	Population		Total (N = 500)
						
					Thai (n = 317)	Burmese (n = 183)	
**Mahidol**	487 G > A	163 Gly > Ser	rs137852314	3	8 (2.5, 38.1%)[5: 3]	25 (13.7, 96.2%) [23: 2]	33(6.6, 70.2%)

**Viangchan**	871 G > A	291 Val > Met	rs137852327	3	4 (1.3, 19.0%)[3: 1]	0 (0, 0)	4(0.8, 8.5%)

**Chinese 4**	392 G > T	131 Gly > Val	rs137852341	3	3 (1.0, 14.3%)[3: 0]	0 (0, 0)	3(0.6, 6.4%)

**Union**	1360 C > T	454 Arg > Cys		2	2 (0.6, 9.5%)[1: 1]	0 (0, 0)	2(0.4, 4.3%)

**Canton**	1376 C > T	459 Arg > Leu	rs72554665	3	2 (0.6, 9.5%)[0: 2]	0 (0, 0)	2(0.4, 4.3%)

**Kaiping**	1388 G > A	463 Arg > His	rs72554664	2	1 (0.3, 4.8%)[1: 0]	1 (0.5, 3.8%)[1: 0]	2(0.4, 4.3%)

**Gaohe**	95 A > G	32 His > Arg	rs137852340	3	1 (0.3, 4.8%)[1: 0]	0 (0, 0)	1(0.2, 2.0%)

**Chinese 3**	493 A > G	165 Asn > Asp	rs137852331	2	0 (0, 0)	0 (0, 0)	0 (0, 0)

**Chinese 5**	1024 C > T	342 Leu > Phe	rs137852342	3	0 (0, 0)	0 (0, 0)	0 (0, 0)

**Coimbra**	592 C > T	198 Arg > Cys	rs137852330	2	0 (0, 0)	0 (0, 0)	0 (0, 0)

**Total**					**21 (6.6,100%) [14: 7]**	**26 (14.2,100%) [24: 2]**	**47 (9.4,100%) [38: 9]**

Variants are identified by their common names with the nucleotide and amino acid positions based on the Genbank sequence with the accession number X03674 and the genomic numbers on sequence with accession number X55448[22]. Data are presented for each variant as numbers of individual positive (population prevalence, prevalence relative to total G6PD positive samples) [n females: n males].

Table 3 shows allele frequency of each of the ten G6PD variants in male and female Thai and Burmese populations. Out of 26 G6PD mutations samples in Burmese population (24 females, two males), G6PD Mahidol was found at a high frequency of 96.2% (25/26), of which 8% (2/25) and 92% (23/25) were hemizygous males and heterozygous females, respectively. Only one heterozygous female sample (3.8%, 1/26) carried G6PD Kaiping variant. The allele frequency of Mahidol variant in males and females were 0.18 and 0.07, respectively (Table 3).

**Table 3:** Allele frequency of G6PD variants in male and female Thai and Burmese populations

Population/Gender (N)	G6PD variant allele frequency						
	
	Mahidol	Viangchan	Chinese 4	Union	Canton	Kaiping	Gaohe
**Thai**							

Male (84)	0.04	0.01	-	0.01	0.02	0.012	-

Female (233)	0.01	0.006	0.006	0.002	-	0.002	0.002

**Burmese**							

Male (11)	0.18	-	-	-	-	-	-

Female (172)	0.07	-	-	-	-	0.003	-

For the Thai population, seven G6PD allelic variants were observed among 21 (14 females, seven males) G6PD mutations samples. The most common genotype was G6PD Mahidol variant [38.1% (8/21)], of which 37.5% (3/8) and 62.5% (5/8) were males and heterozygous females, respectively (Table 1). The allele frequency of Mahidol variant in males and females were 0.04 and 0.01, respectively (Table 1). G6PD Viangchan was found at the frequency of 19.0% (4/21), with 25% (1/4) males (allele frequency 0.01) and 75% (3/4) heterozygous females (allele frequency 0.006). G6PD Chinese 4 was found at the frequency of 14.3% (3/21), with 100% (3/3) heterozygous females (allele frequency 0.006). G6PD Canton was found at the frequency of 9.5% (2/21), with100% (2/2) males (allele frequency 0.02). G6PD Union was found at the frequency of 9.5% (2/21), with 50% males (allele frequency 0.01) and heterozygous females (allele frequency 0.002). G6PD Kaiping and Gaohe were found at the frequencies of 4.8% (1/21), with 100% (1/1) heterozygous females (allele frequencies 0.002 for both variants). No G6PD Chinese 3, Chinese 5, and Coimbra variants were found. No samples were positive for more than one assayed G6PD variant.

Based on results of this limited study sample size, there appeared to be variation in G6PD mutation variants in samples obtained from Thai population residing in different regions. G6PD mutation variants were detected in a total of 17 (17/217: 5.36%), 3 (3/14: 14.28%) and 1 (1/21: 4.76%) from samples collected from the western, central and north-eastern region, respectively. No G6PD variant was found in samples collected from the northern, eastern and southern regions (6 samples for each region). Seven G6PD variants were observed in samples from the west region, of which Mahidol variant was the most predominant (8/17: 47.1%), followed by G6PD Chinese 4 (3/17: 17.6%), G6PD Union (2/17: 11.8%), G6PD Viangchan (1/17: 5.9%), G6PD Gaohe (1/17: 5.9%), G6PD Kaiping (1/17: 5.9%), and G6PD Canton (1/17: 5.9%). The G6PD Viangchan was the only variant found in samples collected from the central region at the frequency of 5.56% (3/14) and G6PD Canton was the only variant found in north-east region at the frequency of 1/21: 4.76%.

## Discussion

Numerous mutations in G6PD gene cause a deficiency of this enzyme in erythrocytes [23]. According to the level of enzyme activity, World Health Organization classified variants of G6PD to five groups: *Class I *(severe deficiency of the enzyme with chronic non-spherocytic > haemolytic anaemia), *Class II *(severe deficiency with enzyme activity < 10% of normal), *Class III *(moderate deficiency with enzyme activity 10-60% of normal), *Class IV *(very mild to none deficiency with enzyme activity 60-100% of normal), and *Class V *(increased enzyme activity) [2,22]. Among the high prevalent variants, G6PD Coimbra, G6PD Chinese 3, G6PD Union, and G6PD Kaiping are classified as Class II variants. G6PD Mahidol, G6PD Viangchan G6PD Chinese 4, G6PD Canton, G6PD Gaohe, and G6PD Chinese 5 are classified as Class III variant [24].

There is the difference in the prevalence and distribution of G6PD gene variants among the Thai and Burmese populations. In Burmese population, four G6PD variants (Mahidol, Coimbra, Union and Canton) were detected. Based on results of our study and those so far published [13], the predominant G6PD variant was G6PD Mahidol, which was found in almost all G6PD variant samples (25/26: 96.2%). Than et al. reported the prevalence of G6PD Mahidol of 17.5% (160/916), while no carriers of the G6PD Viangchan mutation were detected [25]. The study of Nuchprayoon et al. [21] showed 6.7% (12/178) of the Burmese males who lived in southern Myanmar carried G6PD Mahidol variant. Higher diversity of G6PD variants was observed in the Thai population compared to the Burmese population, with seven SNPs assayed being polymorphic and the most commonly identified was G6PD Mahidol. It was noted that, with this limited sample size, no sample positive for more than one assayed G6PD variant was found. Distribution of G6PD-deficient variants also differed among regions. G6PD Mahidol variant (47.1%, 8/17) was dominantly detected in G6PD mutation samples collected from the western region, whereas the Chinese 4, Union, Viangchan, Gaohe, Kaiping and Canton variants were found at low frequencies in this region. G6PD Gaohe, G6PD Chinese 4, G6PD Canton, and G6PD Kaiping are the common variants found in Chinese [26]. Two cases of G6PD Union which is predominantly found in populations in the Philippines [27], and Papua New Guinea [28], were also found in Thai population in this study. These findings support a common ancestry of the population and the theory of genetic drift throughout Southeast Asia.

The results are in agreement with that reported previously by Panich [29] that G6PD Mahidol variant was the major variant in Thai population. The average allele frequency in Thailand is 12%, but with distinct local heterogeneity with increased frequency on the western border particularly in the Mon, Burmese and Karen populations [29]. In another report [11], G6PD Viangchan was found to be the most common mutation (54%), followed by G6PD Canton (10%), G6PD Mahidol (8%), G6PD Kaiping (5%), G6PD Union (2.6%), and G6PD Chinese 5 (2.6%). Among 20 neonates with hyperbilirubinaemia, G6PD Viangchan was also found at the highest frequency (60%), followed by G6PD Canton (10%), G6PD Mahidol, G6PD Union, and G6PD Kaiping (5% each). This variation in the reported prevalence of G6PD variants in Thailand is due to differences in study population, study design (random or selected sample collection), study areas under investigation and techniques used for mutation analysis. This study focused on the investigation of the ten major non-synonymous mutations of G6PD variants, which were reported in Thai and Burmese populations using PCR-RFLP technique. Unlike gene sequencing, PCR-RFLP is very specific for detection of each of the ten G6PD mutation variant and therefore detection of new or other minor mutations could be missing. With this limited sample size, no G6PD mutation variant was found in study samples obtained from the northern, eastern and southern parts of the country. Laosombat [19] reported ten different G6PD variants in a total of 225 G6PD deficient individuals in the southern region (Songkla Province) of Thailand. The three most common variants were G6PD Viangchan (31.3%), G6PD Kaiping (20.1%), and G6PD Mahidol (17.2%). In addition, low prevalence of three additional variants, i.e., G6PD Quing Yuan (G392T), G6PD Mediterranean (C563T), and the new variant, G6PD Songklanakarind (T196A) were identified.

Interestingly, the prevalence of G6PD deficiency and malaria endemicity are thought to be correlated [30]. Several studies suggest that G6PD deficiency provides protection against *P. falciparum *malaria among non-immune adults [30-32]. This association between G6PD and malaria was supported by population genetic analyses of the G6PD locus, which indicated that these mutations may have recently risen in frequency in certain geographic regions as a result of positive selection. According to this observation, prevalence of G6PD deficient variants appeared to be high and variable in the western region of Thailand where malaria incidence is the highest of the country [33]. This may support the link between incidence of G6PD deficiency and malaria endemicity hypothesis [34]. Some previous studies also detected a lower parasite count in G6PD-deficient patients [35-38]. Despite numerous evidences on the protection of *P. falciparum *malaria in G6PD deficiency patients, the high failure rate following treatment with standard anti-malarial regimen is still high [33]. Apart from host genetic factor, parasite factor (e.g., resistant genes) contributes more significantly to treatment response [39]. Recent reports also suggest that the malaria parasite can adapt itself to grow in these variant erythrocytes by producing its own G6PD [40]. The parasite's G6PD enzyme is different to that of humans because it is bifunctional, presenting 6-phosphogluconolactonase (6PGL) activity in the molecule's *N*-terminal region [41]. Its expression in *P. falciparum *is an important factor for parasite survival within the host cell. The host-parasite co-evolutionary processes has been reported to be associated with human's coding gene polymorphisms such as the haemoglobin variants (Hb E, Hb C, Hb S, α- and β-thalassemia), G6PD, membrane receptor (Duffy protein), blood group proteins, HLA (HLA-B53, DRB1*1302), and other immune regulatory region with malaria resistance [42]. Louicharoen et al. [8] has demonstrated that G6PD Mahidol variant has been under strong positive selection for the last 1,500 years and that it reduces *P. vivax *density in humans. The finding provides evidence that *P. vivax *has been a driving force behind the selective advantage conferred by the Mahidol mutation.

In this study with limited sample size, the majority of females were identified as heterozygous carriers of G6PD mutations of all variants. These subjects are also at risk of drug-induced haemolysis if the enzyme activity is sufficiently low. Primaquine, an 8-aminoquinoline, is the prototype of drugs that cause haemolysis in G6PD deficiency. It is the only effective anti-malarial drug to prevent relapses of the persistent liver forms of *P. vivax *and *P. ovale*, as well as to interrupt the transmission of *P. falciaprum *gametocytes. The severity varies considerably among affected individuals with G6PD variants and the dose of primaquine given. The degree of haemolysis in other variants of G6PD exposed to standard primaquine therapy (15-30 mg daily) varies markedly. Daily doses of 15 mg of primaquine for 14 days following a full course of chloroquine when prescribed to Thai G6PD deficient patients where Mahidol variant is predominant, are relatively safe [43]. A single dose of primaquine 45 mg and/or weekly for eight weeks has also been found adequate and safe for the treatment of patients with *P. falciparum *gametocytes and/or *P. vivax *malaria ignoring these red cell G6PD enzyme deficient variants in Myanmar. Administration of primaquine is contraindicated during pregnancy irrespective of the mother's G6PD status [39]. Little is known about the clinical efficacy of anti-malarials in individuals with different G6PD variants. In a more recent study conducted in 2045 samples obtained from six African populations (Burkina Faso, Ghana, Kenya, Nigeria, Tanzania, Mali) with acute uncomplicated falciaprum malaria [44], G6PD genotype or phenotype had no influence on anti-malarial efficacy of combination therapy with artemether-lumefantrine and chlorproguanil-dapsone-artesunate. There is also evidence that anti-malarial drugs in patients with haemoglobinopathies exhibit different pharmacokinetic properties [45,46], and the standard doses of anti-malarials may be less efficacious [47]. For primaquine, there appears to be no difference in the plasma concentrations or pharmacokinetics of primaquine between patients with normal G6PD and G6PD deficiency [48].

Due to the risk of haemolysis in G6PD-deficient individuals, laboratory determination of the patient's G6PD status is recommended before prescribing primaquine. The definitive diagnosis of G6PD deficiency is based on the estimation of enzyme activity, by quantitative spectrophotometric analysis of the rate of NADPH production from NADP [2]. For rapid population screening, several semiquantitative methods have been applied, such as the dye-decolouration test and fluorescent spot tests, for identification of persons who will be at risk from taking primaquine. In most of malaria endemic countries including Thailand, such facilities may be limited. Policy for primaquine administration and optimal dose regimen based on individual patient may not be appropriate, but should be based on endemic area with support of epidemiology data of local G6PD variants. It would be interesting also to examine the haemolytic effect of drugs upon different local variants. Testing of new drugs for haemolytic potential should be done prior to their introduction into areas with high frequencies of the G6PD deficiency.

## Competing interests

The authors declare that they have no competing interests.

## Authors' contributions

KN was involved in providing the conception, design of the study and revised the manuscript critically for intellectual content and approved the final version of the manuscript. RR was involved in the collection of clinical samples. PP, JK, YH, and RY performed the molecular analysis of G6PD variants. PP and WC performed data analysis and interpretation. PP drafted the manuscript. All authors read and approved the final manuscript.
